# “Swab-and-Stain” Nanoparticle Assay for Detection of PBP2a-Expressing Methicillin-Resistant *Staphylococcus aureus* Toward Point-of-Need Environmental Surveillance

**DOI:** 10.3390/microorganisms14091940

**Published:** 2026-09-02

**Authors:** Laura Sutarlie, Sian Yang Ow, Karrie Kwan Ki Ko, Chayaporn Suphavilai, Kar Mun Lim, Patipan Boonsimma, Darren Wei Tan, Niranjan Nagarajan, Xiao Di Su

**Affiliations:** 1 Institute of Materials Research and Engineering (IMRE), Agency for Science, Technology and Research (A*STAR), 2 Fusionopolis Way, Innovis #08-03, Singapore 138634, Singapore; 2 Department of Microbiology, Singapore General Hospital, 20 College Road, Singapore 169856, Singaporedarren.tan.wei@sgh.com.sg (D.W.T.); 3 Genome Institute of Singapore (GIS), Agency for Science, Technology and Research (A*STAR), 60 Biopolis Street, Genome, Singapore 138672, Singapore; suphavilai_chayaporn@a-star.edu.sg (C.S.);; 4 Department of Biochemistry, Yong Loo Lin School of Medicine, National University of Singapore, 10 Medical Drive, Singapore 117597, Singapore

**Keywords:** MRSA, nanosensor, hospital acquired infection, antimicrobial resistant

## Abstract

Surveillance of methicillin-resistant *Staphylococcus aureus* (MRSA) in the environment is crucial for reducing its transmission, particularly in hospitals, nursing homes, and high-contact public areas. Rapid Point-of-Need (PON) tools at healthcare facilities, without involving prolonged bacterial culture in sophisticated laboratories, are needed for environmental surveillance of MRSA. We have developed a gold nanoparticle (AuNP)-based “Swab-and-Stain” assay for rapid and on-site detection of PBP2a-expressing MRSA from environmental surfaces. The assay utilizes anti-PBP2a antibody immobilized on cotton swabs to specifically capture PBP2a-expressing MRSA and anti-MRSA antibody conjugated on AuNPs to detect the captured MRSA on the swabs via sandwiched complex formation. In the presence of PBP2a-expressing MRSA, the swabs were stained in red, as seen with the naked eye. The color intensity of the stain can be quantified by smartphone image analysis for MRSA quantification. Using PBP2a-expressing MRSA samples spiked in real hospital sink and drain matrices, this pilot study demonstrated a “Swab-and-Stain” assay as a proof-of-concept method for detecting MRSA at a concentration as low as 12 CFU/mL. The assay was validated to specifically detect MRSA spiked in real hospital sink and drain matrices. This “Swab-and-Stain” assay provides a rapid, portable, and easy-to-use platform with potential application for PON environmental surveillance of MRSA.

## 1. Introduction

Healthcare-associated infections (HAIs) caused by methicillin-resistant *Staphylococcus aureus* (MRSA) are a major challenge in hospital infection prevention. *Staphylococcus aureus* (*S. aureus*) is a Gram-positive spherical bacterium belonging to the family of Staphylococcaceae. While *S. aureus* frequently exists as a harmless commensal on human skin, in nasal passages, and in the upper respiratory tract, it is also an opportunistic pathogen. Upon breaching human skin or mucosal barriers, it produces a variety of virulence factors capable of causing severe deep-tissue and bloodstream infections [1,2]. *S. aureus* can be classified antigenically into various distinct serotypes based on its capsular and surface polysaccharides, with serotypes 5 and 8 found in 75–80% of clinical isolates [3,4]. Furthermore, due to its genetic diversity, *S. aureus* can be categorized into various clonal complexes and sequence types via multilocus sequence typing.

MRSA refers to a group of *S. aureus* strains that are resistant to methicillin, usually via the *mecA* or *mecC* genes of the staphylococcal cassette chromosome *mec* (SCC*mec*) [5,6]. Standard β-lactam antibiotics (such as penicillins, cephalosporins, and carbapenems) normally kill *S. aureus* by binding to its native penicillin-binding proteins (PBPs) and halting cell wall cross-linking. However, *mecA* encodes an alternative PBP, named PBP2a, whose shielded active site is two to three orders of magnitude less reactive toward β-lactams, allowing continuous cell wall biosynthesis and bacterial survival even under high antibiotic concentrations [7,8]. MRSA was first discovered in hospitals in 1961 and remains on the World Health Organization’s 2024 Bacterial Priority Pathogens List [9]. While MRSA-HAIs outnumber community-acquired infections and account for the majority of invasive MRSA cases [10], several different strains of MRSA have been identified from different genetic lineages as hospital-acquired, community-acquired, and livestock-acquired strains, which are attributed to the evolution of *S. aureus* under selective pressure from the widespread and sometimes irrational use of antibiotics from hospitals to farm settings [6].

Rare strains of MRSA have been reported lacking both the *mecA* and *mecC* genes, showing a form of parallel evolution of antibiotic resistance in *S. aureus* where methicillin resistance is conferred by other genes. These special cases of MRSA include borderline oxacillin-resistant *S. aureus* (BORSA) for antibiotic resistance conferred by *blaZ* hyperproduction [11] and methicillin-resistant lacking *mec* (MRLM) for resistance conferred by altered *pbp4*/*gdpP* genes [12].

Compounding this therapeutic challenge, MRSA is also well-adapted to the hospital environment. It forms robust biofilms on both dry and wet surfaces, tolerates prolonged desiccation, and is continuously shed from colonized patients and hospital staff [5]. Collectively, these properties allow MRSA to establish and maintain environmental reservoirs that drive ward-level transmission, making the environment itself a key target for infection prevention and control (IPC) interventions.

Contaminated high-touch surfaces have been implicated in MRSA hospital outbreaks, and the organism can survive on inanimate surfaces for weeks under typical ward conditions [13,14,15,16,17,18]. In a landmark cohort study, patients admitted to a hospital room whose prior occupant was MRSA-positive had a significantly higher risk of acquiring MRSA themselves [19], and a subsequent intensive care study confirmed that enhanced environmental cleaning eliminates this excess risk and reduces overall MRSA acquisition [20]. Beyond patient rooms, sink drains and P-traps are increasingly recognized as concealed, biofilm-rich reservoirs from which MRSA and other antimicrobial-resistant organisms disperse to patient areas via aerosols and splashes during routine use [21,22]. Although targeted decontamination of such reservoirs reduces MRSA transmission [20], its effectiveness depends on IPC teams knowing where and when contamination is present, and that information is only as timely as the surveillance method used to obtain it.

In current hospital practice, environmental MRSA surveillance relies on laboratory-based workflows that introduce a substantial delay between sampling and results. Swabs collected from the ward are transported to the microbiology laboratory for culture (at least 2 days to complete) or molecular polymerase chain reaction (PCR) analysis (2–4 h turnaround time) [23,24]. These methods are dependent on centralized laboratory infrastructure, leading to delayed results. The delay between swabbing and results means that IPC teams are left to choose between resource-intensive empirical decontamination of broad areas without confirmation or delayed reactive action once laboratory results become available. To close this gap and convert environmental MRSA surveillance from a retrospective audit activity into a near real-time IPC intervention, especially in healthcare facilities without microbiology laboratories, there is a growing demand for Point-of-Need (PON) tools capable of testing environmental samples to deliver rapid results directly at or near the sampling site without relying on laboratory infrastructure [25,26].

Biosensors have emerged as rapid and portable methods to detect microbial pathogens [27,28]. An argonaute–deoxyribonucleic acid enzyme tandem biosensor has been reported for detection of the *nuc* and *mecA* genes of MRSA, with a limit of detection (LOD) of 1 colony-forming unit (CFU)/mL in 65 min [29]. A dual Clustered Regularly Interspaced Short Palindromic Repeats-associated protein 12a (CRISPR-Cas12a) biosensor detected the *mecA* gene at a concentration as low as 1 CFU/mL in 2 h [30]. These MRSA gene-targeting biosensors employ nucleic-acid-based amplification methods to achieve high sensitivity, but they still require multiple steps of nucleic acid extraction and enzymatic reactions, which are not practical for PON. To eliminate tedious nucleic acid extraction steps, biosensors have been developed to target the bacterial cell or specific proteins of MRSA. For example, a nanostructure microfluidic device functionalized with anti-PBP2a antibody was reported for detection of PBP2a-expressing MRSA bacterial cells, with a LOD of 50 CFU/mL in 2 h [31]. A lateral flow assay using bacteriophage cell-wall-binding domain protein-functionalized fluorescence microspheres was reported for MRSA cell detection with a broad linear range of 6.6 × 10^2^–6.6 × 10^7^ CFU/mL in 10 min [32]. Although these biosensors are faster, they require a fluorescence spectrometer or reader for fluorescence emission measurement.

To address the need for PON testing on environmental surfaces, we developed a gold nanoparticle (AuNP)-based “Swab-and-Stain” assay for detection of PBP2a-expressing MRSA cells, the predominant MRSA strain in HAIs. AuNPs, with their inherent plasmonic properties, [33,34] were used to generate a distinct color as a detection signal, which can be observed visually by the naked eye or further quantified by image analysis. AuNP colorimetric bacterial detection has been demonstrated in solution [35] or on membrane filters [36]. We have previously developed a “Swab-and-Stain” assay as a rapid and portable biosensor for detection of *Vibrio* from fish kidney samples [37]. Here, the “Swab-and-Stain” assay was further developed for PBP2a-expressing MRSA detection from environmental surfaces. In this assay, two antibodies targeting either the MRSA cell wall or its PBP2a protein were used, one to modify cotton swabs for capturing MRSA from surfaces and the other to functionalize AuNPs for detecting the captured MRSA cells. We demonstrate the assay on environmental samples from hospital sinks. The “Swab-and-Stain” assay is culture-free, enzyme-free, fast and easy to use, enabling PON environmental surveillance of PBP2a-expressing MRSA.

## 2. Materials and Methods

### 2.1. Materials

HAuCl_4_·3H_2_O (99%), trisodium citrate dihydrate, cetrimonium bromide, protein G (pG) (a high-affinity binding protein to the crystallizable (Fc) region of antibodies), phosphate buffered saline (PBS), Schiff’s reagent, and tryptic soy agar (TSA) were purchased from Sigma-Aldrich (St. Louis, MO, USA). Sterile single-tip cotton swabs with a tip length of 16 mm and a width of 5 mm and eSwabs were obtained from Copan (Brescia, Italy). Mouse anti-MRSA antibody (DS-MB-02323), referred to as Ab1, was purchased from RayBiotech (Peachtree Corners, GA, USA). Mouse monoclonal anti-MRSA PBP2a antibody clone 38 (SAB4200853), derived from hybridomas generated from mice immunized with a purified, recombinant PBP2a surface-exposed region fragment [38], referred to as Ab2, was purchased from Sigma-Aldrich (St. Louis, MO, USA). An Alexa Fluor 488 (AF488) microscale protein labeling kit and TSA blood agar were purchased from Thermo Fisher Scientific (Waltham, MA, USA). An antibody biotin labeling kit was purchased from Abcam (Cambridge, UK).

### 2.2. Bacterial Isolates and Bacterial Culture

*Escherichia coli* (*E. coli*) ATCC 25922, *Pseudomonas aeruginosa* (*P. aeruginosa*) ATCC 15692, *Vibrio parahaemolyticus* (*V. parahaemolyticus*) ATCC 17802, methicillin-susceptible *S. aureus* ATCC 6538, and *mecA*-positive MRSA ATCC 43300 [39] were purchased from ATCC (Manassas, VA, USA).

Methicillin-susceptible *S. aureus* (MSSA) hospital isolates B04224, B04225, and B04226 were obtained from Singapore General Hospital as archived, anonymized clinical isolates originally recovered during routine clinical diagnostics, with species identification and antimicrobial susceptibility phenotype confirmed in accordance with Clinical and Laboratory Standards Institute M100 guidelines [40]. Species identification was performed by matrix-assisted laser desorption/ionization time-of-flight mass spectrometry (Bruker, Billerica, MA, USA). Antimicrobial susceptibility testing was performed and interpreted in accordance with the M100 guidelines [40] and further confirmed by whole-genome sequence analysis using a nanopore sequencing platform from Oxford Nanopore Technologies (Oxford, UK), as detailed in the Supplementary Materials. The assembled genome sequences of the B04224, B04225, and B04226 hospital isolates have been deposited in the National Center for Biotechnology Information BioProject database (under accession number PRJNA1476871) with BioSample IDs SAMN60734694, SAMN60734695, and SAMN60734696, respectively.

All bacteria were cultured on TSA plates at 37 °C. The number of bacterial colonies on the plates after overnight incubation was counted as CFUs. Bacterial suspensions were prepared from fresh overnight bacterial colonies in sterile 1×PBS. Based on multiple bacterial plate count results, a bacterial suspension adjusted to an optical density at 600 nm of 0.25, measured using a spectrophotometer (BioSpec-mini, Shimadzu Corporation, Kyoto, Japan), corresponded to a bacterial concentration of 1–2 × 10^8^ CFU/mL. This bacterial suspension was then serially diluted in 1 × PBS to prepare working concentrations from 10^1^ to 10^7^ CFU/mL. The exact concentration of the prepared bacterial suspension was also confirmed by bacterial plate count.

### 2.3. Verification of Antibody Binding to MRSA

The binding of the antibodies to PBP2a-expressing, *mecA*-positive MRSA ATCC 43300, relative to the negative control bacteria (*E. coli* ATCC 25922 and methicillin-susceptible *S. aureus* ATCC 6538), was first verified by “fluorescence-depletion” tests as described previously [37]. In this assay, each antibody was labeled with a green fluorophore, AF488. The binding of the antibody was measured as the percentage decrease (*%F_decrease_*) of the initial fluorescence intensity of the fluorophore-labeled antibody without bacteria (*F*_control_) relative to the fluorescence intensity remaining in the supernatant after separation of antibody bound on bacterial cells via centrifugation (*F*_sample_). The fluorescence intensity was measured using a fluorescence microplate reader (Tecan Infinite M200, Tecan Group, Männedorf, Switzerland). Details of the “fluorescence-depletion” test are provided in the Supplementary Materials.

The ability of the two antibodies to form a sandwich complex on MRSA cells was further verified by an in-house enzyme-linked immunosorbent assay (ELISA) test. One of the antibodies was coated on a 96-well Maxisorp plate (Thermo Fisher Scientific) as a capture antibody, and the other was labeled with biotin as a detection antibody. MRSA bacterial cells were added into the well coated with the capture antibody, followed by addition of the biotin-labeled detection antibody and subsequent streptavidin–horseradish peroxidase. A washing step was applied between each step to remove unbound species. Sandwich complex formation between the capture antibody, MRSA bacterial cells, and detection antibody was detected as a color change signal from the 3,3′,5,5′-tetramethylbenzidine chromogenic substrate, triggered by the horseradish peroxidase and measured using an absorbance microplate reader (BioTek Synergy 2, BioTek Instruments, Winooski, VT, USA). Further details of the in-house ELISA test and its raw data (Table S1) are provided in the Supplementary Materials.

### 2.4. Preparation and Characterization of MRSA Nanostains

MRSA nanostains were prepared by conjugating Ab1 to pG-coated AuNPs. First, AuNPs with a diameter of 13 nm were synthesized by sodium citrate reduction of HAuCl_4_ [41]. For pG coating, 1.5 mL of 10 nM AuNPs was incubated with 60 µL of 1 mg/mL pG for 30 min, followed by centrifugation at 14,000 *g* for 50 min to remove excess pG. Then, the pG-coated AuNPs were incubated with 180 µL of 50 mg/L Ab1 for 30 min, and deionized water was added to a total volume of 1.5 mL. The Ab1-pG-AuNPs conjugates (shortened as Ab1-AuNPs) were characterized using the absorbance microplate reader (Biotek Synergy 2, BioTek Instruments, Winooski, VT, USA) at 400–800 nm against salt-induced aggregation. Cetrimonium bromide was added to minimize non-specific interactions (final concentration of 3 mg/mL).

### 2.5. Preparation and Characterization of Anti-PBP2a Antibody-Modified Swabs

Anti-MRSA antibody-modified swabs were first prepared by conjugating Ab2 to cotton swabs functionalized with aldehyde silane (triethoxysilylbutyraldehyde) and pG, according to a previously reported method [37]. Briefly, cotton swabs were incubated in 2.5% triethoxysilylbutyraldehyde in ethanol for 2 h, dried in a 90 °C oven for 30 min, and washed with 5 mL of 1×PBS three times. The aldehyde-functionalized swabs were immersed in 5 mL of PBS containing 0.04 μg/mL pG for 2 h and washed three times with 5 mL of PBS. The pG–aldehyde-functionalized swabs were then immersed in 5 mL of 0.5 μg/mL Ab2 solution in PBS, washed three times with PBS, and air-dried.

To characterize the antibody conjugation on the cotton swabs, Schiff’s test for free aldehydes [42] was first used to assess the surface functionality of the swabs at each stage of immobilization (unmodified, after aldehyde silane, after pG, and after Ab2 immobilization). The swabs were dipped in 200 µL of 10% *v*/*v* Schiff’s reagent at room temperature for 15 s. Second, the amount of the antibody adsorbed on each swab was calculated based on the difference in fluorescence intensity of Ab2 labeled with AF488 before and after conjugation of Ab2 to the pG–aldehyde-functionalized swabs. The fluorescence intensity of Ab2 labeled with AF488 (Ab2-AF488) was measured using a fluorescence microplate reader. The fluorescence intensity of Ab2-AF488 at 520 nm was calibrated against various concentrations of Ab2 (0–5 µg/mL). The as-prepared Ab2–pG–aldehyde-modified swabs (shortened as Ab2-swabs) were stored at 4 °C and sterilized under ultraviolet light for 10 min before use.

### 2.6. “Swab-and-Stain” for Detection of MRSA

MRSA was detected by the “Swab-and-Stain” assay using Ab1-AuNPs to stain captured MRSA bacterial cells on Ab2-swabs. The Ab2-swabs could be used to capture MRSA in two ways. First, for experiments with various spiked bacterial suspension samples at known concentrations, the Ab2-swabs were immersed for 1 min in 1 mL of the spiked bacterial suspension. Second, for environmental surfaces, such as the lab drains described in Section 2.7, the Ab2-swabs were used to rub the surfaces directly. The swabs were rotated to ensure maximum coverage. Each Ab2-swab was washed twice with 1 mL of 1% Tween-20 solution to remove environmental particulates and any non-specifically bound materials. After that, 100 µL of the Ab1-AuNP solution was dropped on the swab surface and incubated for 10 min. To remove excess Ab1-AuNPs, each swab was washed with 1 mL of 1% Tween-20 solution.

Images of the two opposite sides (front and back) of the cotton tips of the swabs were taken using a mobile phone camera (Samsung, Suwon, Republic of Korea) in a photo box with a controlled white light-emitting diode. To prevent variation in image resolution and color intensity, the same photo box, light source, and camera settings were used for image capture of all swabs.

For quantification by image analysis, the MRSA nanostain intensity on each swab was measured by Red–Green–Blue analysis in ImageJ v1.54t software (National Institutes of Health, Bethesda, MD, USA). The brightness value was reported as the average intensity, (Red + Green + Blue)/3, of the front and back areas of the cotton tip.

### 2.7. Direct Swabbing of Real Environmental Surfaces

The Ab2-swabs were used directly to swab two sink drains in our biochemistry lab. Each swab was then washed twice in 1% Tween-20 solution before staining with Ab1-AuNPs according to the “Swab-and-Stain” method.

To screen for the presence of MRSA, the same lab sink drains were sampled using sterile cotton swabs. The swabs were first plated on mannitol salt phenol red agar, a selective medium for pathogenic staphylococci. Yellow colonies grown on the mannitol salt phenol red agar plates after incubation at 37 °C for 24 h were then sub-cultured on freshly prepared Mueller–Hinton agar supplemented with 4% NaCl and 4 µg/mL methicillin for 24 h.

### 2.8. Testing of Hospital Environmental Samples

Hospital environmental swabs were collected at Singapore General Hospital (SGH) from inpatient areas, sampling sink and drain surfaces using eSwabs pre-moistened with Amies medium. Following the MetaSUB consortium surface sampling protocol [43], each site was swabbed continuously for 2 min in multiple directions. All swabs were tested for MRSA by selective culture on MRSA Select II chromogenic agar (Bio-Rad, Hercules, CA, USA) at 35 °C. Samples negative for general MRSA on the Select II agar were then combined to generate representative negative matrices containing the background microbiota typical of each surface type in the sampled hospital environment.

A reference MRSA strain (*S. aureus* ATCC 43300 carrying the *mecA* gene, which expresses PBP2a), cultured on MRSA Select II agar and adjusted to 0.5 McFarland units in PBS using a DensiCheck Plus densitometer (bioMérieux, Marcy-l’Étoile, France), was serially diluted into the pooled MRSA-negative matrix to generate spiked samples spanning 10 to 10^6^ CFU/mL. Spike concentrations were confirmed by back-titration on TSA blood agar. Briefly, the bacterial suspension was serially diluted in sterile PBS to a target concentration of approximately 10^3^ CFU/mL. A 0.1 mL aliquot of the designated dilution was spread onto TSA blood agar plates in duplicate, which were then incubated at 37 °C for 24 h. Plates containing 30–300 bacterial colonies were selected for quantification. The initial bacterial concentration was calculated by dividing the colony count by the inoculation volume (0.1 mL) and multiplying the result by the corresponding dilution factor.

## 3. Results

### 3.1. Design of the “Swab-and-Stain” Assay and Antibody Pairing for MRSA

**Figure 1:**
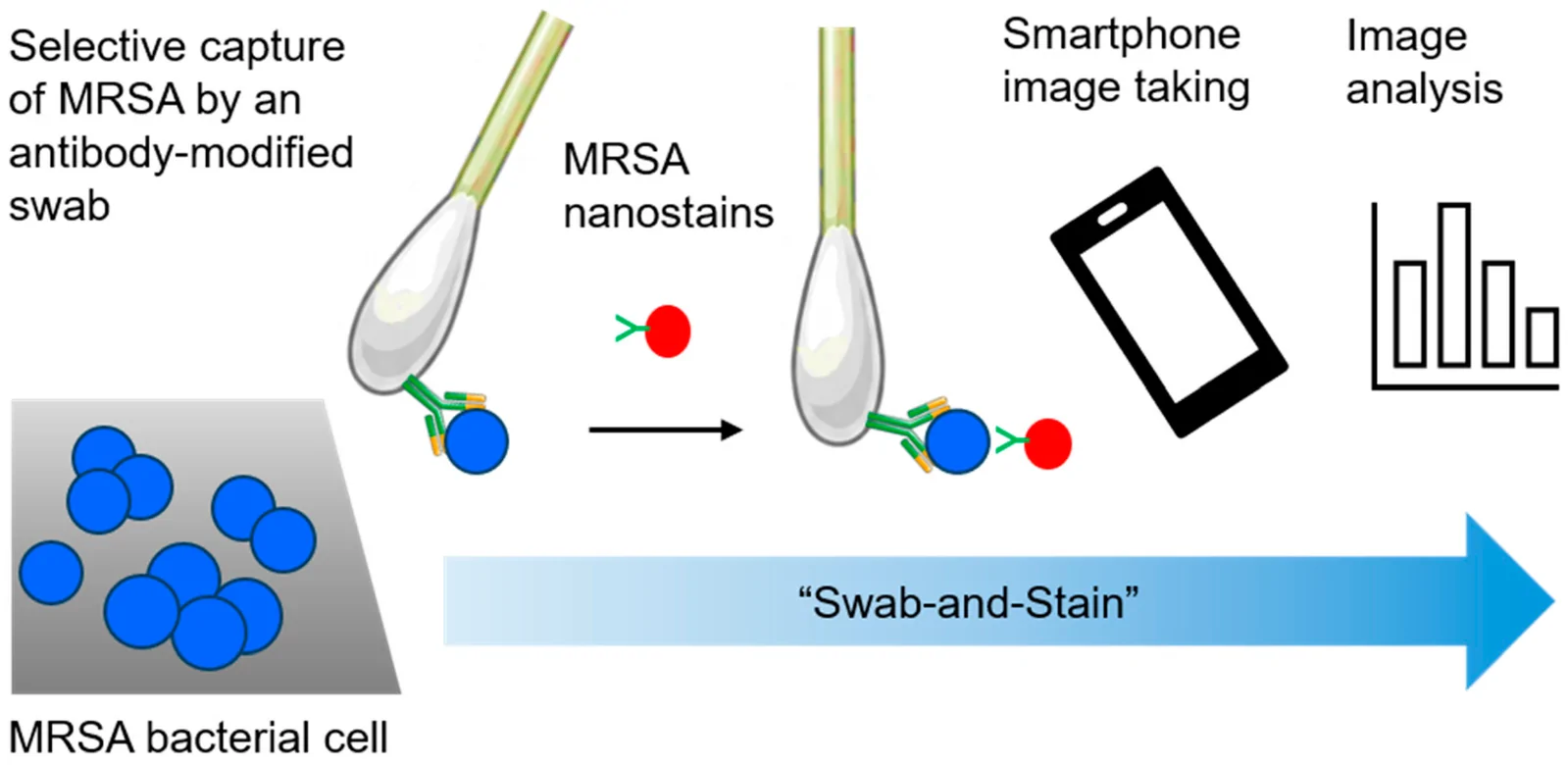
Illustration of the AuNP-based “Swab-and-Stain” assay for detection of MRSA from environmental surfaces. MRSA bacterial cells were selectively captured using an antibody-modified swab. MRSA nanostains made of AuNPs were applied to the swabs to form a sandwich complex on the captured MRSA. The resulting color from the MRSA nanostains on the swabs can be seen by the naked eye or quantified through smartphone image capture and image analysis.

The “Swab-and-Stain” assay was designed for the selective capture of MRSA from surfaces using an affinity-ligand-modified cotton swab, followed by staining of the captured MRSA bacterial cells by affinity-ligand-conjugated AuNPs, resulting in a color signal that can be seen visually and further quantified by smartphone image analysis (Figure 1).

**Figure 2:**
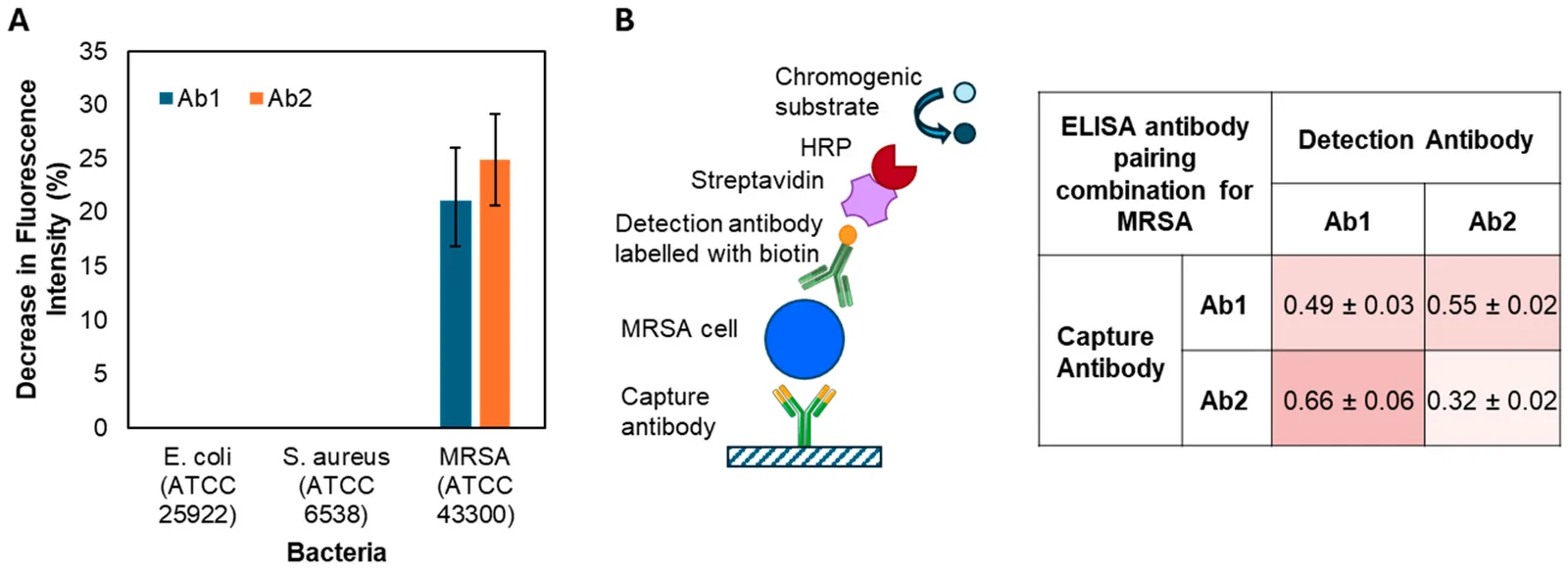
(**A**) Verification of antibody binding to *mecA*-positive MRSA ATCC 43300, relative to *E. coli* ATCC 25922 and methicillin-susceptible *S. aureus* ATCC 6538, by the “fluorescence-depletion” test. The antibodies were fluorescently labeled with AF488. The bar values are the average of at least 3 tests at a bacterial concentration of 10^8^ CFU/mL. (**B**) ELISA test of antibody pairing to form sandwich complexes on captured MRSA, and the respective heatmap of the average and standard deviation of ELISA absorbance at 450 nm for each antibody pairing combination. Higher red color intensity indicates a stronger binding pair.

Antibodies immobilized on the cotton swab and the AuNPs determine the selective recognition and detection of MRSA. We first verified the selectivity of the two antibodies to MRSA bacterial cells by the “fluorescence-depletion” test as a baseline characterization of antibody selectivity to MRSA ATCC 43300 (*mecA*-positive, expressing PBP2a) against two negative control bacteria (*E. coli* ATCC25922 as a representative of Gram-negative bacteria and methicillin-susceptible *S. aureus* ATCC 6538 as a representative of Gram-positive bacteria). In the “fluorescence-depletion test”, fluorophore-labeled affinity ligands bound to bacterial cells were separated from unbound ligands by centrifugation. A decrease in the fluorescence intensity of the free fluorophore-labeled affinity ligands in the supernatant, compared to that without any bacteria, indicated binding to the bacterial cells. Figure 2A shows that both antibodies, i.e., Ab1 (anti-MRSA antibody, RayBiotech, DS-MB-02323) and Ab2 (anti-MRSA PBP2a antibody, Sigma-Aldrich, SAB4200853), had similar *%F_decrease_* values for MRSA but not for the two negative control bacteria. Both antibodies selectively bound MRSA without binding to the two negative controls.

To further confirm that the two antibodies can form a sandwich complex with MRSA bacterial cells, an in-house ELISA assay was performed with the two antibodies taking turns as the capture and detection antibody. Based on ELISA data of possible antibody combinations against MRSA and methicillin-susceptible *S. aureus* ATCC 6538 as a negative control (Figure 2B, Supplementary Materials Table S1), the best antibody pairing, indicated by the highest ELISA absorbance signal, was the combination of Ab1 as the detection antibody and Ab2 as the capture antibody. Hence, Ab1 was used as the detection antibody for conjugation to AuNPs as MRSA nanostains, and Ab2 was used to functionalize the swabs as the capture antibody in the “Swab-and-Stain” test for MRSA.

### 3.2. Antibody Conjugation to AuNPs and Swabs

**Figure 3:**
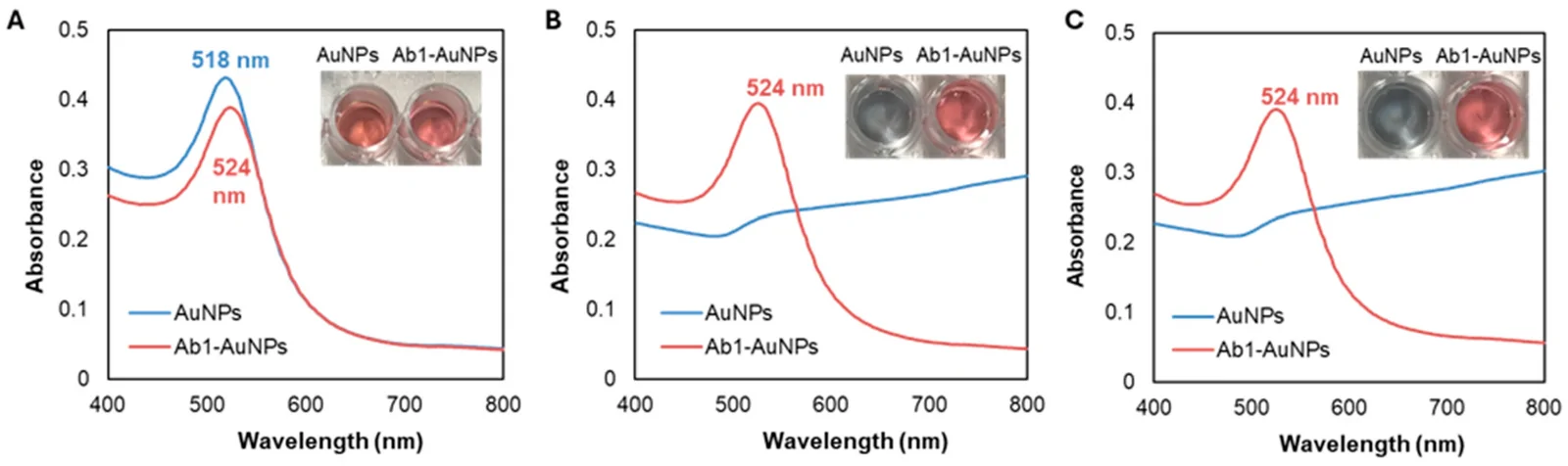
Absorbance spectra of bare AuNPs and Ab1-AuNPs in (**A**) water, (**B**) 0.9% NaCl, and (**C**) 3.5% NaCl. Insets show the corresponding appearance of the AuNPs and Ab1-AuNP solutions in each medium.

Ab1 was conjugated to AuNPs (13 nm in diameter) through pG coating on the AuNPs, followed by Ab1 binding to pG. As pG binds specifically to the crystallizable (Fc) region of an antibody, immobilization via pG ensures that the antigen-binding (Fab) region of the antibody faces outward and allows more antibodies accessible to bind the antigens [44,45]. The successful formation of the Ab1-AuNP conjugate was confirmed first by a shift in the absorption peak of AuNPs from 518 nm to 524 nm with Ab1 (Figure 3A). Second, the Ab1-AuNPs retained their absorbance peak and exhibited higher stability than AuNPs against salt-induced aggregation in the presence of 0.9% NaCl and even at a very high salt concentration (3.5% NaCl), as shown in Figure 3B,C.

**Figure 4:**
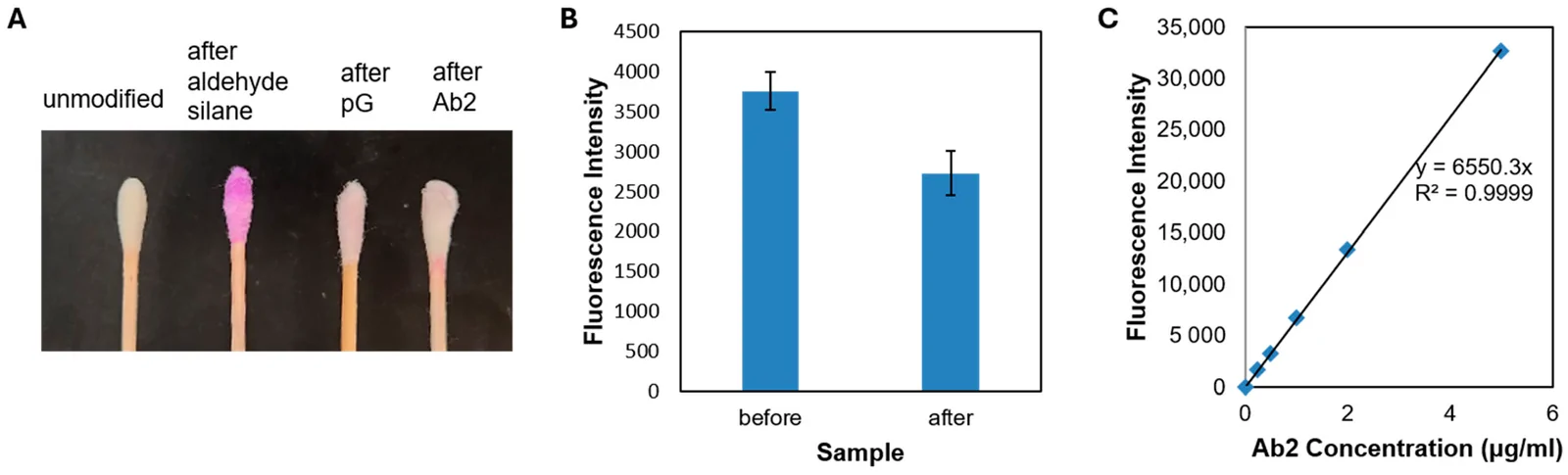
(**A**) Schiff’s reagent test on swabs (unmodified, after aldehyde silane treatment, after pG functionalization, and after Ab2 immobilization). (**B**) Fluorescence intensity of Ab2 labeled with AF488 before and after conjugation to the pG–aldehyde-functionalized swab. (**C**) Calibration of the fluorescence intensity of Ab2 labeled with AF488 at 520 nm as a function of Ab2 concentration.

Ab2 was conjugated to the cotton swabs functionalized with aldehyde silane via pG. To confirm the immobilization of aldehyde, pG, and Ab2 on the swabs, an aldehyde test using Schiff’s reagent was performed on the unmodified swab and on swabs at each stage of immobilization. Figure 4A shows that the unmodified cotton swab remained white, but the swab turned magenta after aldehyde silane treatment. This color change is caused by the reaction of the free aldehyde group on the swab with the Schiff’s reagent [42], indicating successful conjugation of the aldehyde silane to the swab. After pG immobilization on the aldehyde silane-treated swab, the magenta color diminished, presumably due to reaction of the aldehyde group on the swab surface with the amine groups of pG. After Ab2 immobilization, no magenta color was observed on the swab.

To further confirm Ab2 immobilization on the swab, we calculated the amount of Ab2 adsorbed on the swab based on the difference in fluorescence intensity of Ab2 labeled with AF488 before and after conjugation to the pG–aldehyde-functionalized swab (Figure 4B). Based on the calibration (Figure 4C), the Ab2 concentration decreased from 0.57 ± 0.03 µg/mL to 0.42 ± 0.04 µg/mL after conjugation to the pG–aldehyde-functionalized swab. From the Schiff’s aldehyde characterization (Figure 4A), the magenta color corresponding to the free aldehyde group on the swab was lost after pG functionalization, indicating that pG occupied most of the swab surface. As such, the decrease in Ab2 concentration can be attributed mostly to the binding of Ab2 to pG, with minimal non-specific adsorption of Ab2. This decrease suggests that Ab2 was successfully conjugated to the pG-modified swab.

### 3.3. Detection of MRSA Spiked in Clean Buffer Solution

**Figure 5:**
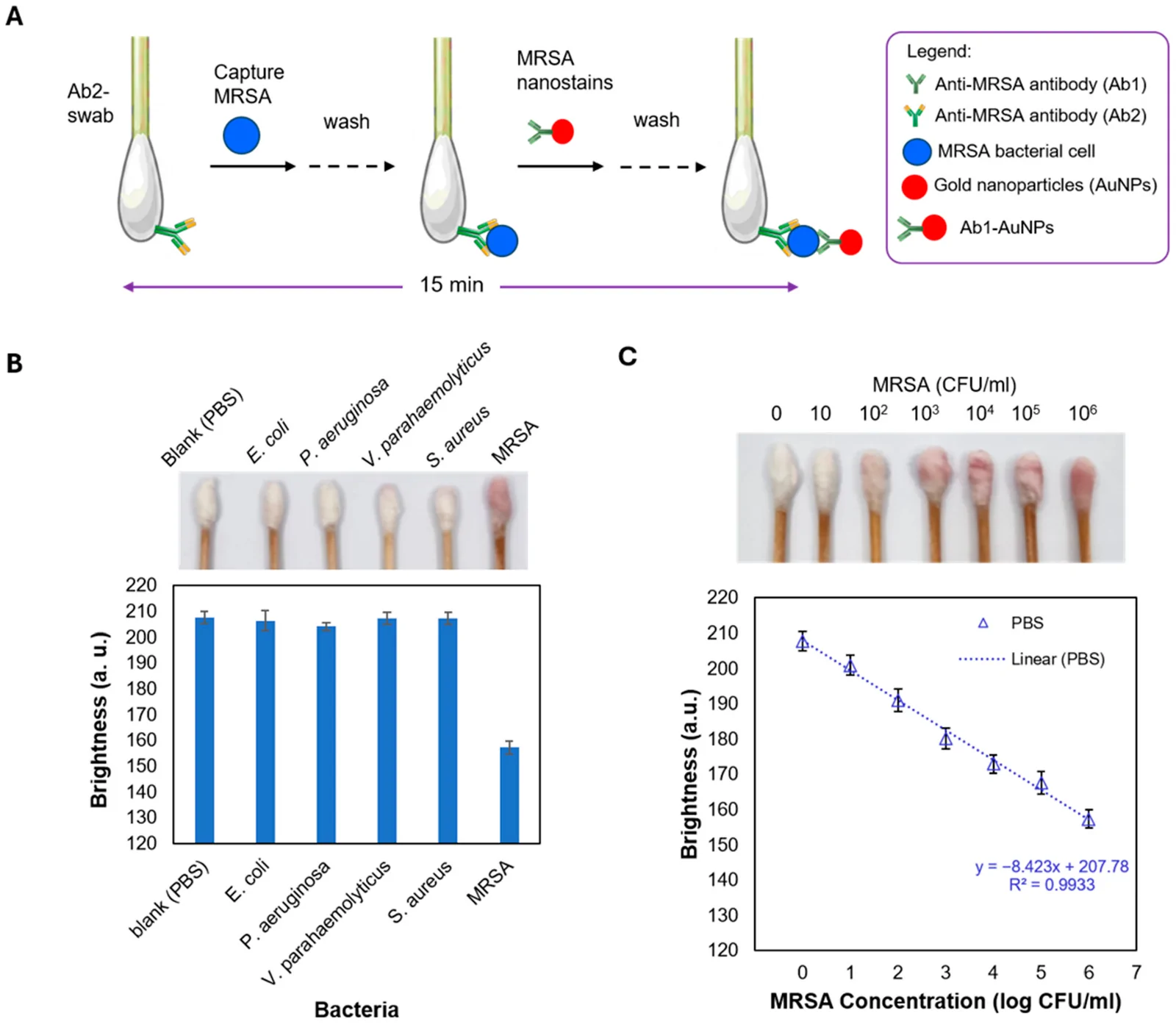
(**A**) Schematic of the “Swabs-and-Stains” method. Swab images and the respective brightness readings for PBS spiked with (**B**) non-MRSA negative control bacteria (*E. coli* ATCC 25922, *P. aeruginosa* ATCC 15692, *V. parahaemolyticus* ATCC 17802, and methicillin-susceptible *S. aureus* ATCC 6538) at 10^7^ CFU/mL and *mecA*-positive MRSA ATCC 43300 expressing PBP2a at 10^6^ CFU/mL (*n* = 3) and (**C**) MRSA ATCC 43300 at increasing concentrations from 0 (no bacteria) to 10^6^ CFU/mL (*n* = 3).

The “Swab-and-Stain” assay was first demonstrated with PBS spiked with *mecA*-positive MRSA expressing PBP2a and with negative control bacteria. In this “Swab-and-Stain” assay (Figure 5A), Ab2-swabs were used to selectively capture PBP2a-expressing MRSA by immersing the Ab2-swabs in the spiked bacterial suspension. The swabs were then stained with the MRSA nanostains (Ab1-AuNPs). The swabs were washed after each step to remove any non-specific binding. When the MRSA-carrying swabs were exposed to Ab1-AuNPs, a red-colored stain could be seen on the swabs due to the formation of a sandwich Ab2/MRSA/Ab1-AuNP complex. The “Swab-and-Stain” assay was tested with PBS spiked with *mecA*-positive MRSA expressing PBP2a at various concentrations and with four negative control bacteria including *E. coli*, *P. aeruginosa*, *V. parahaemolyticus*, and *S. aureus* (methicillin-susceptible). Figure 5B shows that the swabs with the negative control bacteria at 10^7^ CFU/mL remained white, but a dark red stain appeared on the swab with MRSA (10^6^ CFU/mL). From Image J analysis, the brightness value of the negative control swabs was close to the brightness value of the blank swab (PBS, no bacteria), at around 207.7 ± 2.7. The brightness value of the MRSA swab decreased to 157.4 ± 2.6, indicating the selectivity of the “Swab-and-Stain” assay for the PBP2a-expressing MRSA. For various concentrations of MRSA (0–10^6^ CFU/mL), the swabs showed a concentration-dependent reduction in brightness value, with a linear correlation coefficient (R^2^) of 0.9933 (Figure 5C). Based on the average brightness value of the blank (207.7 ± 2.7), with subtraction of 3 times the standard deviation, the MRSA could be detected with an LOD of 12 CFU/mL. In comparison to the “Swab-and-Stain” assay using directly adsorbed Ab1 on AuNPs and Ab2 on aldehyde-modified swabs without pG for antibody orientation, which achieved an LOD of only 78 CFU/mL (Supplementary Materials Figure S1), the “Swab-and-Stain” assay using Ab1-AuNPs and Ab2-swabs via pG-mediated orientation was 6 times more sensitive.

### 3.4. Response to Environmental Surfaces

**Figure 6:**
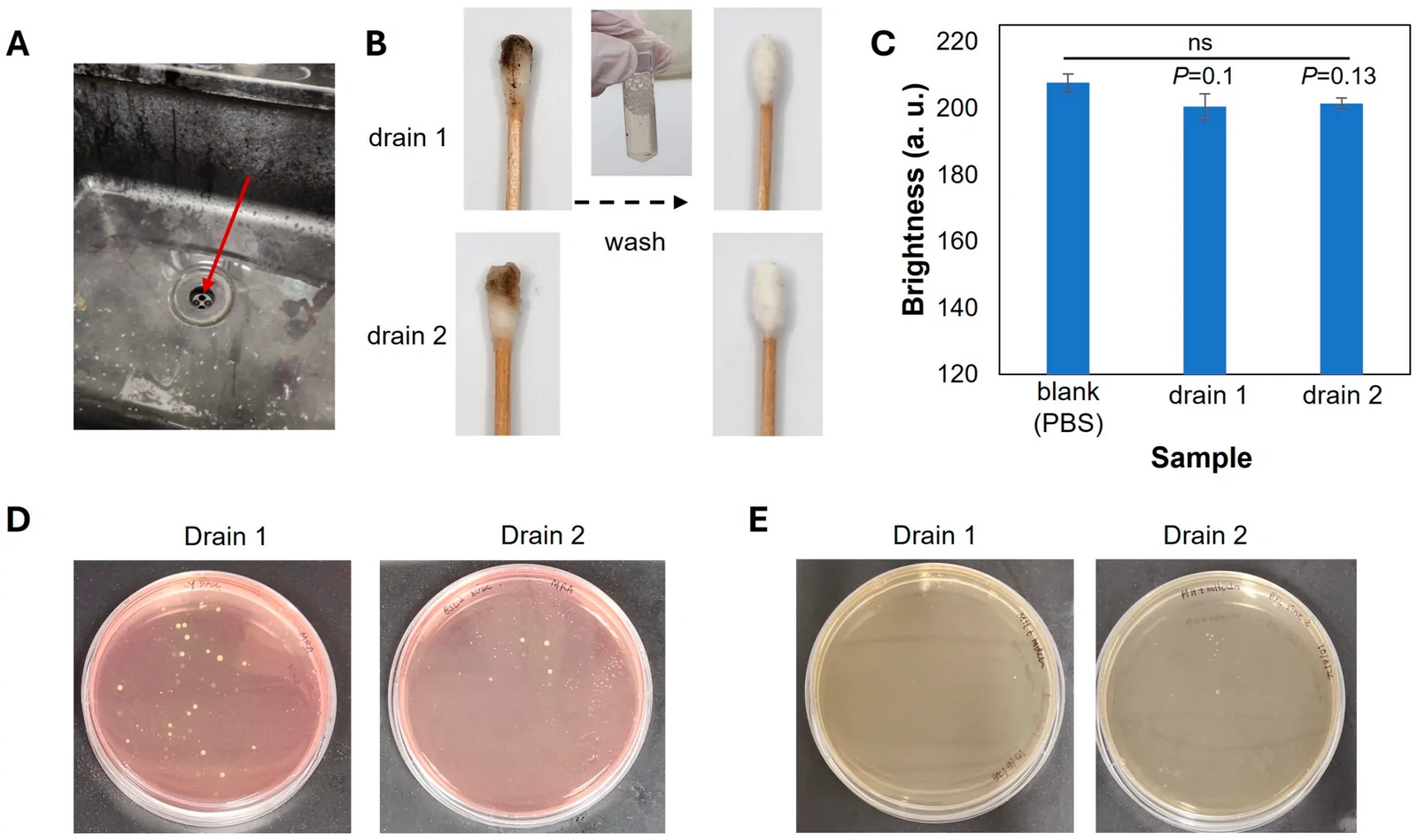
(**A**) Image of one of the sinks. The drain for sampling is marked by the red arrow. (**B**) Images of the drain swabs before and after washing in 1% Tween-20. The dirt from the swab was eluted into the washing solution. (**C**) The brightness value of the drain swabs after staining. Student’s *t*-test: ns = not significant in comparison to blank. (**D**) Bacterial growth on mannitol salt phenol red agar from drain 1 and drain 2 with yellow colonies indicating *Staphylococcus* and other potential mannitol-positive halophiles. (**E**) Sub-culture of the yellow colonies on freshly prepared Mueller–Hinton agar (with 4% NaCl, 4 µg/mL methicillin), indicating no growth of methicillin-resistant bacteria.

To investigate the practicality of the “Swab-and-Stain” assay for environmental sample detection, we used the Ab2-swabs to directly swab the surfaces of two sink drains in our biochemistry lab (Figure 6A). Although black-colored dirt/particulates from inside the sinks attached to the swabs, the majority of them could be removed by thorough washing in 1% Tween-20 (Figure 6B). After applying MRSA nanostains, the brightness value of the sink swabs was not statistically significantly different (*p* > 0.05) from that of the blank swabs (PBS, no bacteria), according to Student’s *t*-test (Figure 6C). We also validated the lab drain samples by bacterial culture, which confirmed that no methicillin-resistant bacteria were found (Figure 6D,E).

### 3.5. Detection of MRSA in Real Hospital Sample Matrix

**Figure 7:**
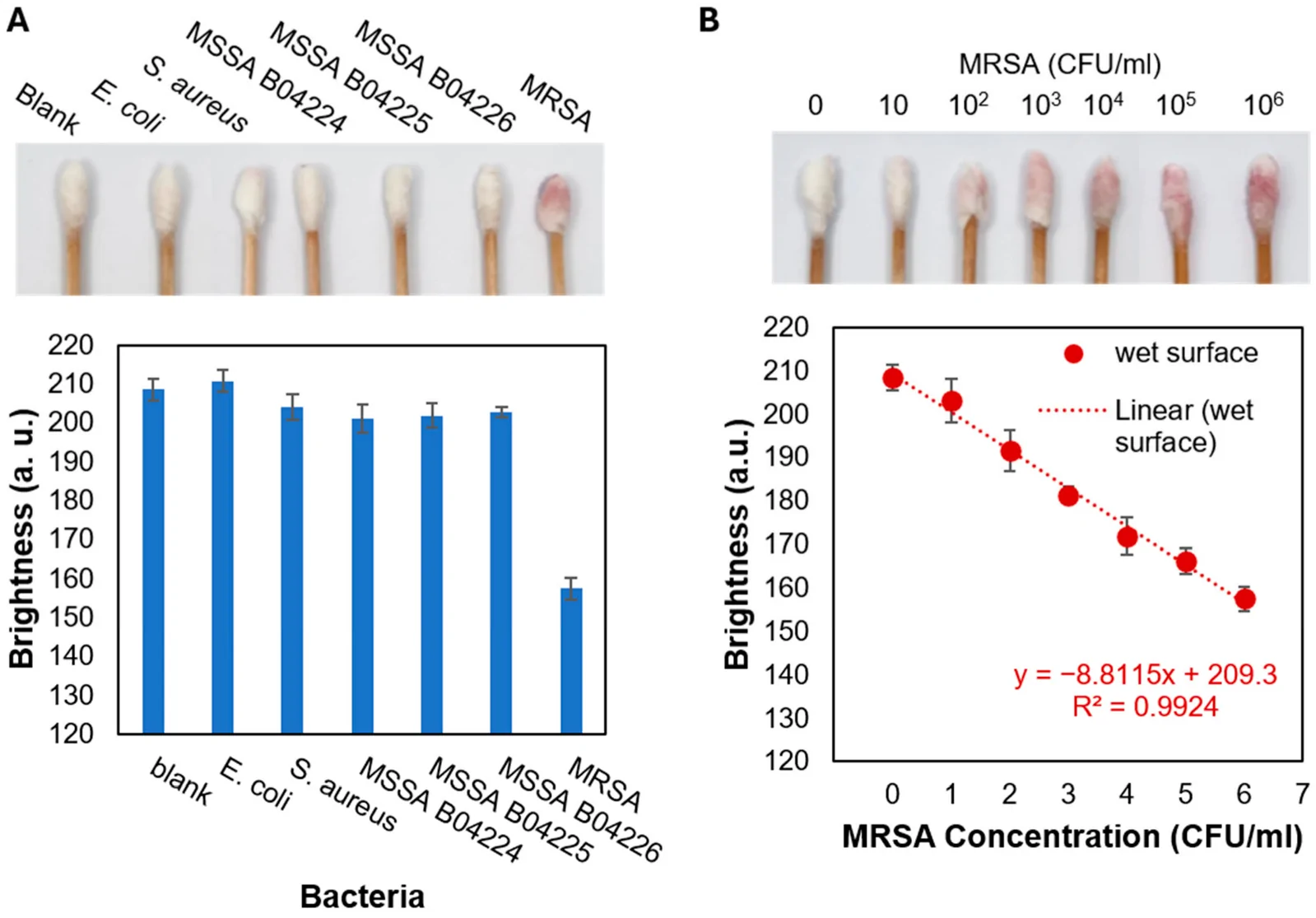
Swab images and the respective brightness readings for (**A**) various negative control bacteria and MSSA hospital isolates and (**B**) MRSA at increasing concentrations from 0 (blank, no bacteria) to 10^6^ CFU/mL (*n* = 3), spiked into the real hospital sample matrix.

Hospital swab samples were collected from a few sinks in inpatient areas and tested by PCR. As no MRSA strains were found in these hospital samples by culture testing, for the purpose of our study, *mecA*-positive MRSA ATCC 43300 expressing PBP2a was spiked into PCR-negative eluate from hospital sink swabs. In addition, the “Swab-and-Stain” assay was also tested against two negative control bacteria (*E. coli* ATCC 25922 and methicillin-susceptible *S. aureus* ATCC 6538) and three MSSA hospital isolates (B04224, B04225, and B04226) spiked into the real hospital sample matrix. In this real hospital sample matrix containing the background microbiota typical of a hospital environment, a significant decrease in the brightness value was observed only on the MRSA swab (Figure 7A). No red stain was observed on the rest of the swabs, including the blank (hospital sample matrix without bacteria spiking), the negative control bacteria (*E. coli* ATCC 25922, *S. aureus* ATCC 6538), and the three MSSA hospital isolates. The “Swab-and-Stain” assay remained selective for PBP2a-expressing MRSA in the real hospital sample matrix.

The degree of the brightness decrease on the PBP2a-expressing MRSA swabs was proportional to the concentration of MRSA (Figure 7B). The linear correlation of the brightness value to MRSA concentration in the hospital sample matrix had a gradient (y = −8.8115x + 209.3, R^2^ = 0.9924) very close to that of the linear correlation in PBS (y = −8.423x + 207.78, R^2^ = 0.9933). The LOD of the “Swab-and-Stain” assay for the real hospital sample matrix was also 12 CFU/mL, based on the average brightness value of the blank with subtraction of 3 times the standard deviation, similar to what was observed in clean PBS. The real hospital sample matrix did not interfere with the selectivity or the LOD.

## 4. Discussions

The results demonstrate a “Swab-and-Stain” assay for the detection of PBP2a-expressing MRSA based on sandwich complex formation on MRSA cells using Ab1 (anti-MRSA antibody)-conjugated AuNPs and Ab2 (anti-PBP2a antibody)-functionalized swabs. Although no prior studies have reported the binding selectivity of Ab1, we evaluated its selectivity using a fluorescence-depletion assay against PBP2a-expressing MRSA and a panel of negative control bacteria. Furthermore, the selectivity of the “Swab-and-Stain” assay does not depend on Ab1 alone but also on Ab2, which has previously been validated for selective recognition of PBP2a [38]. The results in Section 3.3 and Section 3.5 also demonstrate selective detection of MRSA spiked in both PBS and a hospital environmental matrix, against negative control bacteria including *E. coli*, methicillin-susceptible *S. aureus* (ATCC 6538), and three MSSA hospital isolates. While PBP2a is expressed in the majority of MRSA strains, variation in PBP2a expression level and the emergence of less common MRSA strains, including strains expressing the divergent PBP2c homolog, BORSA, and MRLM, should be considered. Antibodies with broader recognition of various MRSA strains, or co-functionalization with multiple antibodies recognizing different MRSA strains, should be explored for future development of the “Swab-and-Stain” assay.

The “Swab-and-Stain” assay can detect PBP2a-expressing MRSA at concentrations as low as 12 CFU/mL in PBS when the antibodies on the AuNPs and swabs are immobilized via pG-mediated functionalization, which controls the orientation of the antibodies. The same LOD of 12 CFU/mL was achieved for PBP2a-expressing MRSA spiked in the real hospital environmental matrix. The environmental background does not interfere with detection, even when directly swabbing surfaces (as demonstrated on lab drains in Section 3.4), as the environmental particulates and background matrices can be removed through the swab washing steps.

Table 1 compares the “Swab-and-Stain” assay with commercial PCR and other MRSA detection methods in terms of the required sample pretreatment, sample-to-result turnaround time, LOD, and equipment involved.

In comparison to detection methods targeting nucleic acids, while the “Swab-and-Stain” assay has a 12 times higher LOD than the argonaute–DNAzyme [29] and the dual CRISPR-Cas12a biosensor [30], it achieves a 12 times lower LOD than commercial PCR [46]. The “Swab-and-Stain” assay also has a fast assay time (15 min), which is significantly faster than these methods (2 h). In addition, unlike these methods, the “Swab-and-Stain” assay only uses a smartphone camera, without tedious sample treatment. Compared to other biosensors targeting bacterial proteins, the “Swab-and-Stain” assay achieved an LOD 4 times lower than the microfluidic device, which requires a lab-based fluorescence microscope [31], and 55 times lower than the lateral flow assay [32]. This practical “Swab-and-Stain” assay is suitable for on-site PON application in environmental surveillance of MRSA.

The current study is a proof of concept, which has only been tested with a limited number of PBP2a-expressing MRSA and MSSA strains spiked in a real hospital environmental sample matrix. Future studies with a larger panel of MRSA and MSSA strains and larger prospectively collected environmental samples will be important to further validate the assay’s performance before deployment. Given that there are many other antimicrobial-resistant pathogens, the “Swab-and-Stain” assay has the potential to be further customized for the surveillance of other antimicrobial-resistance species.

## 5. Conclusions

We developed an AuNP-based “Swab-and-Stain” assay for specific detection of PBP2a-expressing MRSA from environmental surfaces. It has a rapid sample-to-result turnaround time of 15 min, with an LOD of 12 CFU/mL for quantitative detection by smartphone image analysis. It was demonstrated using PBP2a-expressing MRSA spiked in a real hospital sample matrix (from sinks and drains). The “Swab-and-Stain” assay in this pilot study shows potential as a PON test for the on-site detection and quantification of PBP2a-expressing MRSA. With further comprehensive validation, it may reduce delays toward timely environmental surveillance of MRSA.

## Figures and Tables

**Table 1:** Comparison of “Swab-and-Stain” and other methods for MRSA detection.

Method	Target	Sample Pretreatment	Sample-to-Result Turnaround Time	LOD (CFU/mL)	Equipment Involved	Ref.
Commercial PCR (BD Max MRSA XT)	*SCCmec-orfX* junction, *mecA* and *mecC* genes	Automated cell lysis, nucleic acid extraction	2 h	150	BD Max automated extraction and thermocycling	[46]
Argonaute-DNAzyme tandem detection	*Nuc* and *mecA* genes	Cell lysis, nucleic acid extraction, isothermal amplification	65 min	1	Incubator, microplate reader	[29]
Dual CRISPR-Cas12a biosensor	*mecA* gene	Cell lysis, nucleic acid extraction	2 h	1	Incubator, microplate reader	[30]
Nanostructured microfluidic device	PBP2a	No	2 h	50	Fluorescence microscope	[31]
Lateral flow assay	Cellular wall binding sites of MRSA bacteriophage	No	10 min	660	Fluorescence strip reader	[32]
“Swab-and-Stain” assay	PBP2a	No	15 min	12	Smartphone	This study

## Data Availability

The raw data supporting the conclusions of this article will be made available by the authors on request.

## Supplementary Materials

The following supporting information can be downloaded at: https://www.mdpi.com/article/10.3390/microorganisms14091940/s1, Figure S1: Image of the swabs and the corresponding brightness readings using directly adsorbed Ab1 on AuNPs and Ab2 on aldehyde-modified swabs for PBS spiked with MRSA ATCC 43300 at increasing concentration from 0 (no bacteria) to 10^3^ CFU/mL; Table S1: Absorbance at 450 nm from ELISA testing of various antibody pairings.

## Abbreviations

The following abbreviations are used in this manuscript:

AF488	Alexa Fluor 488
AuNPs	gold nanoparticles
BORSA	borderline oxacillin-resistant *Staphylococcus aureus*
CFU	colony-forming unit
CRISPR-Cas	Clustered Regularly Interspaced Short Palindromic Repeats and CRISPR-associated protein
ELISA	enzyme-linked immunosorbent assay
HAIs	healthcare-associated infections
IPC	infection prevention and control
LOD	limit of detection
MRLM	methicillin-resistant lacking *mec*
MRSA	methicillin-resistant *Staphylococcus aureus*
MSSA	methicillin-susceptible *Staphylococcus aureus*
PBP	penicillin-binding protein
PBS	phosphate buffered saline
PCR	polymerase chain reaction
PON	point-of-need
pG	protein G
TSA	tryptic soy agar
USA	United States of America
