# Supplementary material for: “Swab-and-Stain” Nanoparticle Assay for Detection of PBP2a-Expressing Methicillin-Resistant Staphylococcus aureus Toward Point-of-Need Environmental Surveillance
Source: Microorganisms. 2026 Sep 2;14(9):1940. doi: 10.3390/microorganisms14091940 (PMC13609530; doi:10.3390/microorganisms14091940)
Supplement: Supplementary file 1 [file microorganisms-14-01940-s001.zip › microorganisms-4439736-supplementary.pdf]

Supplementary Materials

"Swab-and-Stain" Nanoparticle Assay for Detection of PBP2a-Expressing Methicillin-Resistant *Staphylococcus aureus* Toward Point-of-Need Environmental Surveillance

Laura Sutarlie [1,*], Sian Yang Ow [1], Karrie Kwan Ki Ko [2], Chayaporn Suphavilai [3], Kar Mun Lim [3], Patipan Boonsimma [3], Darren Wei Tan [2], Niranjan Nagarajan [3,4] and Xiao Di Su [1]

[1] Institute of Materials Research and Engineering (IMRE), Agency for Science, Technology and Research (A*STAR), 2 Fusionopolis Way, Innovis #08-03, Singapore 138634, Singapore
[2] Department of Microbiology, Singapore General Hospital, 20 College Road, Singapore 169856, Singapore; darren.tan.wei@sgh.com.sg (D.W.T.)
[3] Genome Institute of Singapore (GIS), Agency for Science, Technology and Research (A*STAR), 60 Biopolis Street, Genome, Singapore 138672, Singapore; suphavilai_chayaporn@a-star.edu.sg (C.S.)
[4] Department of Biochemistry, Yong Loo Lin School of Medicine, National University of Singapore, 10 Medical Drive, Singapore 117597, Singapore
* Correspondence: laura_sutarlie@a-star.edu.sg

Nanopore Sequencing

A single colony of each hospital isolate was selected and inoculated into 5 ml of sterile brain heart infusion medium at 37°C, 200 rpm overnight. The culture was centrifuged at 8,000×g for 5 min, and the supernatant was discarded. The pellet was then lysed in 500 µl of ATL buffer added with lysing matrix E for 10 min and centrifuged at full speed for 5 min. The supernatant (200 µl) was incubated with 4 µl of 100 mg/ml RNase A at room temperature for 2 min, followed by incubation with 25 µl of proteinase K at 56°C for 20 min. Bacterial DNA in the treated supernatant was extracted using AMPure XP bead purification (Beckman Coulter, Brea, CA, USA). AMPure XP beads (250 µl, 1×) were added to the treated supernatant and incubated for 5 min. The magnetic beads were placed on a magnetic rack, and the supernatant was discarded. The collected magnetic beads were washed with 1 ml of 70% v/v ethanol, air dried, and eluted in the elution buffer. The extracted DNA was run on a 0.5% agarose gel to check the DNA integrity. DNA fragments above 1 kb was selected for Nanopore sequencing (Oxford Nanopore Technologies, Oxford, UK).

Raw Oxford Nanopore Technologies sequencing reads were filtered and trimmed using Chopper v0.5.0 to remove low-quality reads. Read statistics were calculated using SeqKit v2.5.1 and NanoStat v1.6.0. Taxonomic classification of sequencing reads was performed using Kraken2 v2.1.2 with the PlusPF-16 database (release version 2025-07-14). Genome assembly was conducted using Flye v2.9.1-b1780 with default parameters. The resulting assemblies were polished using Medaka v1.11.1 to improve consensus sequence accuracy. Assembly quality was assessed using QUAST v5.0.2, and genome annotation was performed using the National Center for Biotechnology Information Prokaryotic Genome Annotation Pipeline (PGAP; release version 2025-05-06.build7983). Antimicrobial resistance (AMR) genes were identified using AMRFinderPlus v4.2.7 with database release 2026-06-15.1. The AMR predictions were reported based on matches within the AMRFinderPlus reference database.

Fluorescence-Depletion Test

First, each of the antibodies (Ab1 and Ab2) was labeled with Alexa Fluor 488 (AF488) fluorescent dye using a microscale protein labeling kit purchased from Thermo Fisher Scientific (Waltham, MA, USA), according to the manufacturer's instruction. In the "fluorescence-depletion" test, 50 µL of 0.02 mg/ml antibody (Ab1 or Ab2) labeled with AF488 was mixed with 400 µl of 10^8 CFU/ml bacterial suspension and 50 µl of 10×PBS. The mixture was incubated for 30 min with moderate shaking at room temperature. The antibody bound on the bacterial cells were separated from the unbound one by centrifugation for 30 min at 14,000×g. The fluorescence intensity of the unbound antibody (F_{sample}) was measured from 100 µl of the supernatant using a fluorescence microplate reader (Tecan Infinite M200, Tecan Group, Männedorf, Switzerland) under excitation at 470 nm. Similar experiment was performed without bacterial cells to measure the initial fluorescence intensity of the fluorophore-labeled antibody in the absence of bacteria ($F_{control}$). The percentage decrease in fluorescence intensity ($\%F_{decrease}$) due to antibody binding to bacterial cells was calculated as:

$$\% F_{decrease} = [\, (F_{control} - F_{sample}) \,/\, F_{control} \,] \times 100\% \quad (1)$$

Enzyme-Linked Immunosorbent Assay (ELISA)

Four combinations of the two antibodies to form sandwich complexes on MRSA cell were verified by an in-house ELISA test. Duplicate wells of 96-well plates were coated with 1 µg/ml Ab1 or Ab2 in carbonate-bicarbonate buffer (100 µl per well) and incubated at 4 °C overnight. The wells were washed with a washing buffer (0.05% Tween-20 in PBS) three times. Bovine

serum albumin (5% in the washing buffer) was added to block the wells, which were then incubated at 37 °C for 1.5 hours. Similarly, the wells were washed three times with 0.05% Tween-20 in PBS. Then, 100 µl of 10^6 CFU/ ml MRSA was added into each well and incubated for 1 hour at 37 °C. After washing three times, 100 µl of 1 µg/ml biotin-labeled Ab1 or Ab2 (as the detection antibody) and incubated for 1 hour at 37 °C. Following washing, sandwich complex formation by the biotin-labeled antibody on the captured MRSA was detected by adding 100 µl of 1 µg/ml streptavidin-horseradish peroxidase into each well, and incubating for 1 h at 37 °C. After washing, to generate the colorimetric signal, 50 µl of one-step 3,3',5,5'-tetramethylbenzidine was added to each well. Bound horseradish peroxidase in the wells changed the 3,3',5,5'-tetramethylbenzidine color from transparent to blue. After 10 min incubation, 50 µl of 1 M hydrochloric acid was added to each well to stop the reaction. The absorbance of the solution in each well was measured using an absorbance microplate reader (Biotek Synergy 2, BioTek Instruments, Winooski, VT, USA) at wavelength of 450 nm.

Raw data from the ELISA for MRSA ATCC 43300 and the negative control methicillin-susceptible *S. aureus* ATCC 6538 are shown in Table S1.

Table S1. Absorbance at 450 nm from ELISA testing of various antibody pairings.

Capture antibody	Detection antibody			
	Ab1		Ab2	
	S. aureus (ATCC 6538)	MRSA (ATCC 4300)	*S. aureus* (ATCC 6538)	MRSA (ATCC 4300)
Ab1	0.357	0.52	0.244	0.568
	0.278	0.467	0.333	0.541
Ab2	0.305	0.619	0.289	0.329
	0.324	0.709	0.341	0.301

"Swab-and-Stain" Assay using Directly Adsorbed Ab1 on AuNPs and Ab2 on Aldehyde-Modified Swabs without pG

To determine whether pG is required for the immobilization of antibodies on the AuNP and the swabs, we conjugated Ab1 to AuNPs and Ab2 to aldehyde-modified swabs through direct adsorption without the intermediate pG layer to control antibody orientation. Conjugates of directly adsorbed Ab1 on AuNPs were prepared by incubating 1.5 mL of 10 nM as-synthesized AuNPs with 180 µl of 50 mg/l Ab1 (the same amount of Ab1 added to pG-coated AuNPs) for 30 min. The resulting conjugates prepared through direct adsorption aggregated when PBS was added to a final concentration of 1×PBS. To stabilize the conjugates against salt-induced aggregation, 30 µl of 100 mg/l thiolated polyethylene glycol (MW 850) was added, and the mixture was incubated for another 30 min. Excess thiolated polyethylene glycol was removed through centrifugation at 8,000×g for 20 min. Meanwhile, Ab2 was directly adsorbed on aldehyde-modified swabs by immersing the swabs in 5 ml of 0.5 µg/ml Ab2 solution in PBS. The swabs were then washed with PBS three times and immersed in 5 ml of 0.4 mg/ml BSA for 2 h to block any free aldehydes, followed by washing with PBS three times and air-drying.

Figure S1 shows that lighter red stains can be observed on the swabs. From Image J analysis, the gradient of the brightness value in correlation to MRSA concentration is less steep (−4.9) than that of the pG-mediated "Swab-and-Stain" assay (−8.4). The LOD of the "Swab-and Stain" assay prepared from directly adsorbed antibodies is 78 CFU/ml based on the average brightness value of the blank minus 3 times the standard deviation.

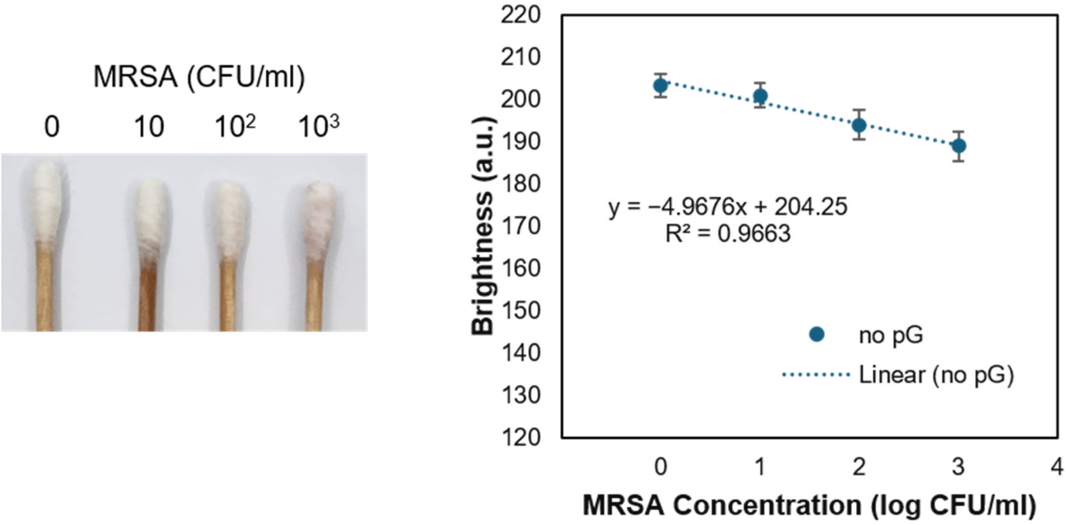

Figure S1. Image of the swabs and the corresponding brightness readings using directly adsorbed Ab1 on AuNPs and Ab2 on aldehyde-modified swabs for PBS spiked with MRSA ATCC 43300 at increasing concentration from 0 (no bacteria) to 10^3 CFU/ml.
